# de Quervain's Disease among Patients Visiting the Orthopaedic Outpatient Department of Tertiary Care Centre: A Descriptive Cross-sectional Study

**DOI:** 10.31729/jnma.7947

**Published:** 2023-01-31

**Authors:** Poojan Kumar Rokaya, Dhan Bahadur Karki, Tufan Singh Kathayat, Mangal Rawal, Rachit Sharma, Anil Ghimire

**Affiliations:** 1 Department of Orthopedics and Trauma Surgery, Karnali Academy of Health Sciences, Jumla, Karnali, Nepal; 2 Nepal Orthopedic Hospital, Jorpati, Kathmandu, Nepal

**Keywords:** *de Quervain's disease*, *surgery*, *tenosynovitis*

## Abstract

**Introduction:**

de Quervain's disease is one of the common causes of wrist pain. It can cause serious disability and absence from work due to impaired functioning of the wrist and hand. The aim of this study was to find out the prevalence of de Quervain's disease among patients visiting the orthopaedic outpatient department of a tertiary care centre.

**Methods:**

This was a descriptive cross-sectional study conducted among patients visiting the orthopaedic outpatient department of a tertiary care centre after receiving ethical approval (IRC KAHS Reference: 078/079/56). This study was conducted from 1 January 2021 to 30 December 2021 from hospital medical records. A convenience sampling method was used. Patients with de Quervain's disease from 16 to 60 years were included in this study. Clinically diagnosis of de Quervain's disease was based on the tenderness of the radial styloid process, tenderness over the first extensor compartment on resisted thumb abduction or extension and positive Finkelstein test. Point estimate and 95% Confidence Interval were calculated.

**Results:**

Out of 9600 orthopaedic outpatients, de Quervain's disease was seen in 128 (1.33%) (2.684.52, 95% Confidence Interval).

**Conclusions:**

The prevalence of de Quervain's disease was similar when compared to other studies conducted in similar settings.

## INTRODUCTION

de Quervain's disease is one the most common cause of wrist pain and disability encountered. It is characterized by myxoid degeneration, mucopolysaccharide accumulation and thickening of tendon sheath leading to stenosing tenosynovitis of the abductor pollicis longus (APL) and extensor pollicis brevis (EPB) tendon.^1^ It is triggered by overuse but can occur spontaneously in middle-aged women.^2,3^

Management is usually conservative consisting of analgesics, immobilisation and local steroid injection. Initial treatment with steroid injection may yield complete pain relief in over 70% of patients. However, 30% have chronic pain despite two doses of steroid injections.^4^ Operative treatment is preferred for such refractory cases when repeated steroid injection does not respond or when there are persistent symptoms after six weeks of conservative treatment. Operative management is considered a simple procedure and involves release of the first dorsal compartment of wrist.^5^

The objective of this study was to find out the prevalence of de Quervain's disease among patients visiting the orthopaedic outpatient department of a tertiary care centre.

## METHODS

This study was a descriptive cross-sectional study done after obtaining ethical approval from Institutional Review Committee (IRC KAHS Reference: 078/079/56). This is a descriptive cross-sectional study conducted at Karnali Academy of Health Sciences, Nepal. All the patients with the diagnosis of de Quervain's disease presenting to the outpatient department of orthopaedics from 1 January 2021 to 30 December 2021 were identified through medical records. A convenience sampling technique was used. The sample size was calculated by using the following formula:


$$\begin{array}{ll} \text{n} & ={\text{Z}}^{2}\times \frac{\text{p}\times \text{q}}{{\text{e}}^{\text{2}}}\\ & =1.96^{2}\times \frac{0.50\times 0.50}{0.02^{2}}\\ & =2401 \end{array}$$

where,

n = required sample size
Z = 1.96 at 95% confidence interval (CI)
p = prevalence taken as 50% for maximum sample size calculation
q = 1-p
e = margin of error, 2%

The calculated sample size was 2401. Doubling the sample size, the total sample size was 4802. However, a 9600 sample size was taken. Clinically diagnosed patients of de Quervain's disease (tenderness of radial styloid process, tenderness over the first extensor compartment on resisted thumb abduction or extension and positive Finkelstein test), age group of 16-60 years were included in the study. Exclusion criteria were patients with other pathologies in the wrist, skin lesions (scabies, eczema), cervical radiculopathy, previous fracture around the wrist, and patients with diabetes mellitus, gout and rheumatoid arthritis.

All the relevant information including patient history and pre-operative and post-operative findings were recorded in individual structured patient proforma. Patients were initially managed with analgesics, intra-lesional steroid injection, immobilization of the affected wrist with a splint and physiotherapy. The surgical release was performed in those patients in whom two doses of steroid injection did not respond or when there were persistent symptoms after six weeks of conservative treatment. Written informed consent was obtained and no extra financial burden was given to the patient. Elective surgery was performed after the necessary pre-operative workup. Surgeries were performed in the operation theatre by council-registered orthopaedic surgeons. Patients were followed up clinically at two weeks, three months and six months. Post-operative clinical assessment was performed by Finkelstein test, visual analogue scale (VAS) score and modified mayo wrist score.^6-8^ In Finkelstein's test the affected thumb is bent into the palm, fingers are closed over the thumb making a fist and finally, the wrist is bent ulnarly. This test reproduces the patient's pain in its characteristic location over the first compartment of the extensor retinaculum. Finkelstein stated that the EPB and APL tendons and the tendon sheath were stretched in this position.^6^ Modified mayo wrist score is interpreted as excellent (90-100), good (80-89), fair (65-79) and poor (<65).^9^

Data were entered in a Microsoft Excel Version 2016 and analysed with IBM SPSS Statistics 20.0. Point estimate and 95% CI were calculated.

## RESULTS

Out of 9600 orthopaedic outpatients, de Quervain's disease was seen in 128 (1.33%) (1.10-1.56, 95% CI). The mean age of patients was 40.2±12.84 years. There were 115 (90%) females and 13 (10%) males. The right side was involved in 72 (56.25%) and the left side in 56 (43.75%). In this study, 102 (79.68%) were right-hand dominance and 26 (20.31%) were left-hand dominance. The surgical release was performed in 30 (23.43%) refractory cases among 128 (100%) patients of de Quervain's disease. Out of 30 refractory cases, 16 (53.33%) presented with symptoms of 6-12 months duration, 4 (13.33%) with less than six months duration and 10 (33.3%) with more than one-year duration. A total of 20 (66.66%) were housewives, 6 (20%) were students, 3 (10%) were job holders and 1 (3.33%) was a painter among 30 (23.43%) refractory de Quervain's disease patients (Table 1).

**Table 1:** Postoperative VAS score (n= 30).

Duration	(Mean±SD)
2 weeks	2.1±0.56
3 months	1.43±2.10
6 months	1.13±1.73

At six months follow up, 27 (90%) patients had excellent, 1 (3.33%) had good and 2 (6.66%) had fair results. We observed duplication of APL tendons in 5 (16.66%) patients. The complication was noted in 2 (6.66%) patients who had persistent pain over the first dorsal compartment which was managed with analgesics. The mean duration of surgery was 12±7.34 minutes with a range of 10-25 minutes. All patients returned to normal activities within the mean duration of two weeks after surgery.

**Table 2:** Postoperative modified mayo wrist score (n= 30).

Duration	(Mean±S.D.)
2 weeks	87.5±1.78
3 months	93±1.68
6 months	93.33±0.54

## DISCUSSION

The prevalence of de Quervain's disease was 1.33% among patients visiting the orthopaedic outpatient department of tertiary care hospital over a period of one-year duration. A similar prevalence rate is reported in other studies in the literature.^4,5^ de Quervain's disease can cause serious disability and absence from work due to impaired functioning of the wrist and hand. Many conservative treatment modalities have been described such as heat, cold, strapping, splints, rest, and massage. Local anaesthetic, corticosteroid injection and oral analgesics have been described. When conservative treatment implemented for six weeks fails, operative treatment is considered.^10^ In this study, the female-to-male ratio was 9:1 which is similar to previous studies.^11,12^ The higher incidence of de Quervain tenosynovitis in women could be due to biological hormonal effects and overexposure to biomechanical repetitive work-related constraints.^13^ The mean age of patients in this study was 40.2±12.84 years. de Quervain's tenosynovitis was common in similar age groups in the literature.^10,14^ This shows that de Quervain's tenosynovitis is a disease of people of a productive age group. Hence, it has a serious economic impact. We observed dominant hand involvement in 79.68% of the patients. However, a study conducted in Iran found 80 % of involvement in the non-dominant hand.^15^ Altogether, 20 (66.67%) out of 30 patients of refractory de Quervain's disease were housewives. Likewise, all the female patients were housewives exposed to manual work in a similar study done in Pakistan.^11^ The complication was noted in two patients (6.66%) who had persistent pain over the first dorsal compartment which was managed with analgesics. A three-part retrospective study done at Yale University School of Medicine reported severe recurrent wrist pain, wrist weakness, scar tenderness, numbness and tingling at the site of surgery as complications.^16^ During the surgical procedure, different anatomical variations of the first extensor compartment were found. Five patients had duplication of the APL tendon. An Australian study reported that EPB tendon was in a separate compartment in 10 cases out of 79 wrists in 71 patients.^17^ Similarly, Japanese authors observed that 16 patients had a septum in the first extensor compartment and six patients had a single canal.^18^ Such variation could lead to possible misinterpretations and failure to decompress the EPB tendon resulting in the recurrence of symptoms. VAS score was 1.13 at six months follow-up in this study. The post-operative VAS score in this study is comparable to other studies with surgical release of refractory de Quervain's disease.^10,19,20^ Modified mayo wrist score was 93.33 at six months follow-up in our study. Very few studies have evaluated the functional outcome after surgical release of refractory de Quervain's disease using a modified Mayo wrist score.

This was a non-comparative study with a small sample size. The pain was evaluated by the VAS score which is a subjective score. Our findings represent the prevalence of a single tertiary care centre in Karnali province. A multicentric comparative study with large sample size and longer follow-up duration should be conducted to generalize the findings.

## CONCLUSIONS

The prevalence of de Quervain's disease was similar when compared to other studies conducted in a similar setting. The majority of the patients improve with conservative treatment. Surgical release of de Quervain's disease can be considered in those patients who do not respond to conservative treatment for up to six weeks.
